# Melioidosis Presenting as Retropharyngeal Abscess

**DOI:** 10.31662/jmaj.2025-0267

**Published:** 2025-09-12

**Authors:** Yong Wang Su, Aziah Ab Rani, Chee Yik Chang

**Affiliations:** 1 Department of Medicine, Hospital Sultanah Aminah, Johor, Malaysia; 2 Department of Otorhinolaryngology, Hospital Sultanah Aminah, Johor, Malaysia; 3 Infectious Disease Unit, Department of Medicine, Hospital Sultanah Aminah, Johor, Malaysia

**Keywords:** Melioidosis, *Burkholderia pseudomallei*, retropharyngeal abscess

A 36-year-old man with underlying diabetes mellitus presented with a 2-week history of left-sided neck pain, fever, and dysphagia. Physical examination revealed a left intraoral lesion discharging pus. Incision and drainage of the lesion were performed, yielding approximately 10 cm^3^ of purulent material. A contrast-enhanced computed tomography (CT) scan of the neck revealed bilateral retropharyngeal collections (Figure 1). CT thorax and abdomen revealed no abnormalities. Pus culture grew *Burkholderia pseudomallei*, which was susceptible to ceftazidime, meropenem, and trimethoprim-sulfamethoxazole. Blood cultures were negative. The patient was treated with intravenous ceftazidime 2 g every 6 hours for 2 weeks, followed by oral trimethoprim-sulfamethoxazole for eradication therapy upon discharge.

**Figure 1.**
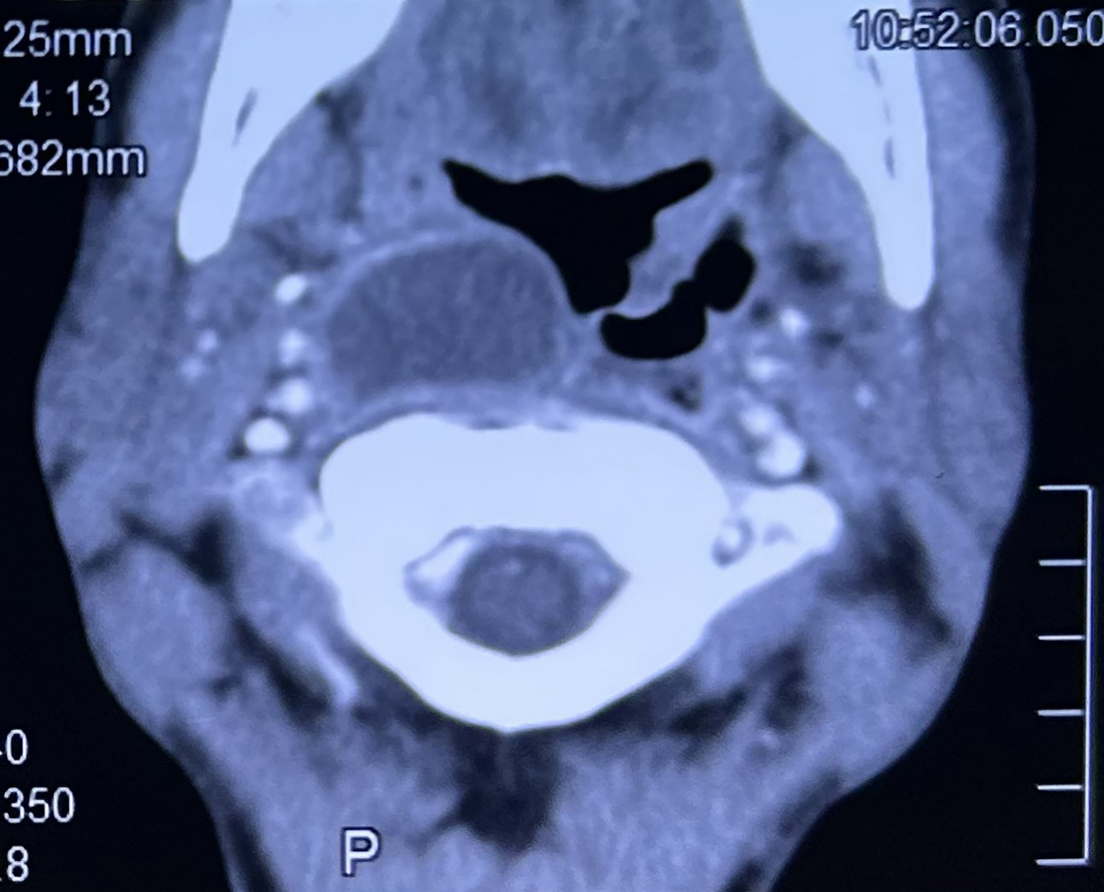
CT scan of the neck showing bilateral retropharyngeal collections extending from the nasopharynx to the hypopharynx. The collection on the right measured 1.7 × 3.0 × 5.1 cm, whereas the left measured 2.5 × 1.6 × 5.3 cm and demonstrated the presence of air locules. CT: computed tomography.

Deep neck space infections are serious because of airway and vital structure involvement. Although usually polymicrobial, *B. pseudomallei* is a rare cause. Melioidosis is a tropical disease caused by *B. pseudomallei* ^(1)^. Retropharyngeal abscess caused by melioidosis is exceptionally rare. In addition to appropriate antimicrobial therapy, appropriate imaging and surgical drainage of the abscess is an important part of management ^(2)^.

## Article Information

### Author Contributions

All authors were involved in the acquisition of data, drafting the article, and final approval of the version to be submitted. Chee Yik Chang contributed to the conception and design of the study and supervision.

### Conflicts of Interest

None

### Consent Statement

Written consent has been obtained from the patient to publish the information, including the photographs.
